# Epidemiological, clinical and hematological profiles of visceral leishmaniasis among patients visiting Tefera Hailu Memorial Hospital, Northeast Ethiopia: a 4 year retrospective study

**DOI:** 10.1038/s41598-023-28139-5

**Published:** 2023-01-17

**Authors:** Habtu Debash, Habtye Bisetegn, Marye Nigatie, Getu Abeje, Daniel Getacher Feleke

**Affiliations:** 1 Department of Medical Laboratory Sciences, College of Medicine and Health Sciences, Wollo University, Dessie, Ethiopia; 2 Department of Medical Laboratory Sciences, College of Health Sciences, Woldia University, Woldia, Ethiopia; 3 Department of Biomedical Science, College of Medicine and Health Sciences, Samara University, Samara, Ethiopia; 4 Department of Microbiology, Immunology and Parasitology, College of Health Sciences, Addis Ababa University, Addis Ababa, Ethiopia

**Keywords:** Microbiology, Diseases

## Abstract

Visceral leishmaniasis is a major, life-threatening parasitic disease that still remains a serious public health problem in Ethiopia. Understanding the epidemiological, clinical, and hematological profiles of visceral leishmaniasis patients is important for implementing evidence-based control strategies. It is also important for early treatment and to decrease the mortality rate from the disease. Therefore, this study was aimed at assessing the epidemiological, clinical, and hematological profiles of visceral leishmaniasis among patients visiting Tefera Hailu Memorial Hospital, Northeast Ethiopia. A retrospective study was conducted at Tefera Hailu Memorial Hospital from September 2017 to August 2021. Data were collected from the medical records of suspected patients who were tested by the rK39 rapid diagnostic by strictly following standard operating procedures. The data was summarized using Microsoft Excel and analyzed using SPSS 26 version software. Descriptive statistics were used to describe the epidemiological, clinical, and hematological profiles of visceral leishmaniasis patients. A p-value < 0.05 was considered statistically significant. The overall positivity rate for visceral leishmaniasis was 23.4% (132/564). The result of this study indicated a fluctuating yet declining trend in VL over the past 4 years. From a total of 132 VL confirmed cases, the numbers of cases were highest among males (78.0%), those 15–29 years of age (37.1%), and urban residents (89.4%). Furthermore, Abergele (11.0%), Sehala (6.0%), and Ziquala (5.0%) districts had the highest number of VL cases. The major clinical presentations of patients were fever (96.2%), splenomegaly (94.7%), and general weakness (80.3%). With regard to hematological profiles, the most common findings were anemia (86.4%), thrombocytopenia (81.8%), leucopenia (78.8%), neutropenia (74.2%), and pancytopenia (71.2%). In the study area, the VL positivity rate was high. Our findings also concluded that VL causes significant alterations in clinical and hematological parameters. Therefore, the zone health office and other concerned stakeholders should strengthen evidence-based control programs for VL.

## Introduction

Leishmaniasis is a neglected tropical disease caused by obligate intracellular protozoan parasites of the genus *Leishmania*. It is transmitted to humans by the bite of infected female sandflies^1^. Visceral leishmaniasis affects the spleen, liver, bone marrow, and lymph nodes and is caused by the *Leishmania donovani* complex^2^. It is a chronic, systemic disease characterized by fever, hepatosplenomegaly, lymphadenopathy, pancytopenia, weight loss, weakness, and, if untreated, death^3^. Secondary bacterial infection, severe anemia, malnutrition, severe absolute neutropenia, severe thrombocytopenia, a higher neutrophil count, liver injury, kidney failure, and disseminated intravenous coagulation are the main syndromes of visceral leishmaniasis. Bacterial infection and bleeding were mutually exclusive events leading to death^4,5^. Red blood cell (RBC) sequestration and destruction in an enlarged spleen, immunological processes, and alterations in RBC membrane permeability in VL patients are all possible causes of anemia^6^.

Visceral leishmaniasis is endemic in 78 countries but mainly affects economically disadvantaged populations^7^. About more than 90% of the global burden of VL cases was reported from Bangladesh, Brazil, India, Kenya, Somalia, South Sudan, Sudan, and Ethiopia^8^. In Ethiopia, an estimated 3.2 million people are at risk of VL, and 3700–7400 cases occur annually^9^. Visceral leishmaniasis is a growing health issue in Ethiopia that is constantly expanding to new areas. Ethiopia's northern lowlands and southern regions have previously been reported to have the largest number of VL cases^10,11^.

The geographic distribution of leishmaniasis is determined by the sand fly species that act as vectors, their ecology, and the rate of multiplication of leishmania parasites inside the vector^12^. Over the last few years, Ethiopia has performed a lot of activities in the prevention and control of leishmaniasis, like sandy fly vector control, the use of long-lasting insecticide-treated nets, and the early diagnosis and treatment of cases^10^. However, challenges remain, such as instability, population movements, environmental changes, political commitments, and impairment of immunity due to HIV/AIDS and malnutrition^13,14^. In Ethiopia, leishmaniasis transmission is often seasonal and highly variable due to differences in topography, rainfall patterns, and transmission. Therefore, it is necessary to update the epidemiological information on leishmaniasis, its effects, and the vulnerabilities of poor communities, as well as the development of a new 2030 road map for neglected tropical diseases (NTDs) in the context of the global sustainability agenda^15^.

The epidemiological, clinical, and hematological profile of visceral leishmaniasis patients in the area has not been given documented. Therefore, examining the VL positivity rate in various settings has paramount importance for developing area-specific evidence-based interventions, making informed decisions, and tracking the effectiveness of VL management strategies. Furthermore, the disease's fatality in relation to clinical and hematological anomalies has received insufficient attention. Hence, there is a scarcity of baseline data on VL in Ethiopia, particularly in relation to clinical and hematological abnormalities. The purpose of this study was to look at the epidemiological, clinical, and hematological profiles of visceral leishmaniasis in patients who visited Tefera Hailu Memorial Hospital, in Northeast Ethiopia, over the last 4 years (2017/18–2020/21).

## Methods

### Study design, area and period

**Figure 1:**
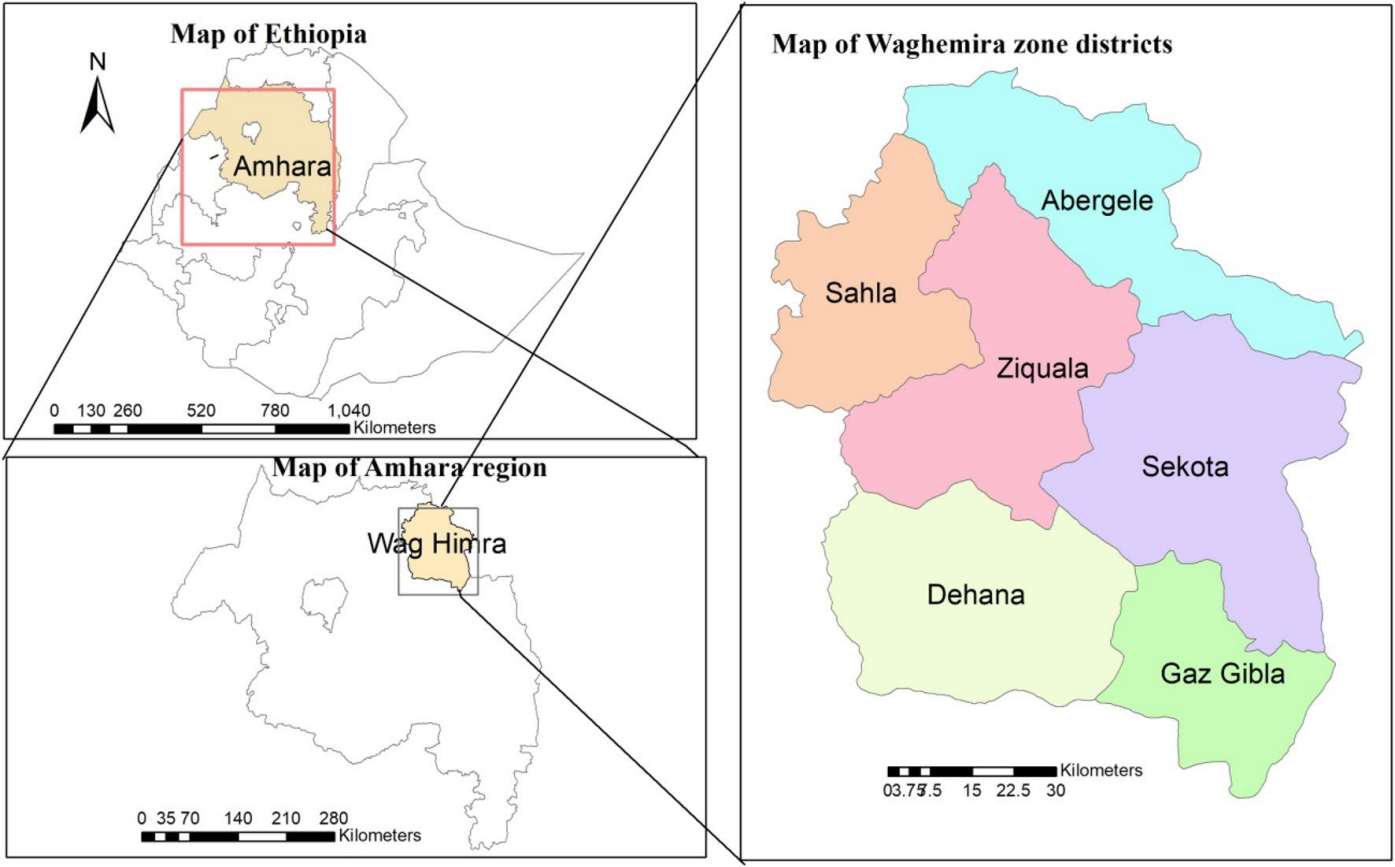
Map of the study area. The above maps were generated by ourselves using the ArcGIS version 10.8 software. The shape files of Ethiopia's administrative regions, zones, and woredas were freely downloaded from a link https://africaopendata.org/dataset/ethiopia-shapefiles.

A 4-year (September 2017 to August 2021) retrospective study was conducted by reviewing laboratory registration books from Tefera Hailu Memorial Hospital. Tefera Hailu Memorial Hospital is located in Sekota town, which is at a distance of 720 kms from Addis Ababa, Ethiopia’s capital. The population of Waghemra zone was reported to be 463,505 people in the 2007 Ethiopian census^16^. The hospital is one of the leishmaniasis diagnostic and therapeutic centers in the Amhara region of northeast Ethiopia. The visceral leishmaniasis diagnosis in the hospital was performed according to the WHO guidelines and following standard operating procedures^17^. Its service is also extended to nearby or border zones, such as the border areas of the North Gondar zone and the Tigray region (Fig. 1). The study participants were patients who had been suspected of having a visceral leishmaniasis infection and tested positive for the recombinant K39 test during their diagnosis.

### Data collection and procedure

Over a 4-year period (September 2017 to August 2021), secondary data were collected from the patient’s medical records. Variables such as the total number of clinically suspected and confirmed cases in each month, year, and socio-demographic characteristics such as age, sex, and residence were collected. In the study hospital, clinically suspected individuals were diagnosed with VL using a fresh peripheral whole blood sample that was tested by the rk39 with a sensitivity of 88.0% and a specificity of 84.0% in Ethiopia^18^. Patients meeting the VL clinical case definition should be tested by the rK39 rapid diagnostic test (RDT), followed by the direct agglutination test (DAT), or tissue aspiration if the RDT is negative^19^. Otherwise, RDT-positive patients should be started on VL treatment. However, no DAT test was available at the study health facility. Furthermore, VL patients were also screened for other infectious diseases like malaria, mycobacterium tuberculosis, hepatitis virus, HIV, and more.

Blood was collected and analyzed by a Mindray BC-5800 hematology analyzer. The clinical and hematological profiles of VL patients were recorded using a well-prepared data collection sheet from their medical charts. The data was collected by experienced medical laboratory technologists using a data collection sheets. The data were checked for accuracy, completeness, and consistency before analysis. Any incomplete data, like the result, age, sex, residence, and date of examination, were excluded from the analysis.

### Data management and statistical analysis

Microsoft Excel was used to summarize data extracted from laboratory logbooks and medical records. Then, using the SPSS version 26 software package, data was entered and analyzed. Descriptive statistics were employed to calculate frequencies and percentages of overall VL prevalence and trends of VL transmission in terms of seasons, years, sex, age, and patient place of residence. A chi-square test was used to compare the association of VL burden by sex, age groups, occupation, and residence. Kolmogorov and Shapiro–Wilk tests were used to check the distribution of continuous variables. Because hematological parameters were normally distributed, the mean and standard deviation were used to present the value of each parameter. A p-value ≤ 0.05 was considered statistically significant. Finally, the findings were summarized using tables, line graphs, and bar charts.

### Ethical approval

The study utilized existing secondary data from medical records at Tefera Hailu Memorial Hospital and did not require ethical approval or consent. This was waived by the Ethical Committee of the College of Medicine and Health Sciences at Wollo University. A supportive letter was obtained from the department of Medical Laboratory Sciences, College of Medicine and Health Sciences, Wollo University. Permission to use the data and conduct the study was obtained from the Waghemra Zone Health Department and Tefera Hailu Memorial Hospital. No patient details that may link to the patient’s identity, like names, were used, and confidentiality was maintained. The data was also analyzed only for the intended purpose, and the results were communicated in an aggregated manner to the hospital and zone health office. All methods were carried out in accordance with the Declaration of Helsinki guidelines and regulations.

## Results

### The overall positivity rate of VL cases

**Figure 2:**
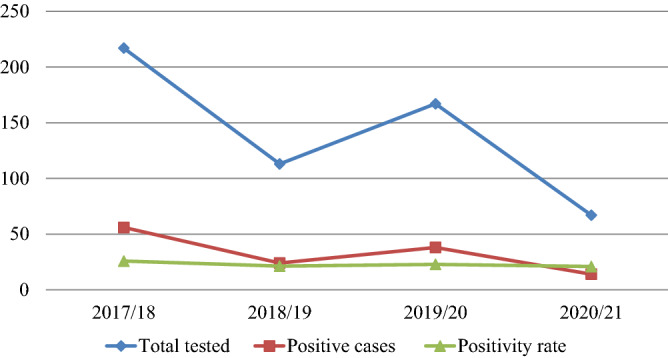
Trends of VL positivity rate at Tefera Hailu Memorial Hospital, Sekota, Northeast Ethiopia from January 2017 to December 2021.

A total of 564 VL-suspected patients were requested for laboratory confirmation analysis during the last 4 years, from September 2017 to August 2021. Out of the total VL-suspected cases, 132 were positive, for an overall positivity rate of 23.4%. According to the findings of this study, an average of 33 VL-confirmed cases was reported in each year. Of the total 132 VL-confirmed patients, the maximum number of cases reported was 25.8% (56/217) in the year 2017/18. On the other hand, the minimum number of cases 20.9% (14/67) was recorded in 2020/21. In general, the result of this study indicated a fluctuating yet declining trend in VL over the past 4 years (Fig. 2).

### Monthly and seasonal variation of VL cases

**Figure 3:**
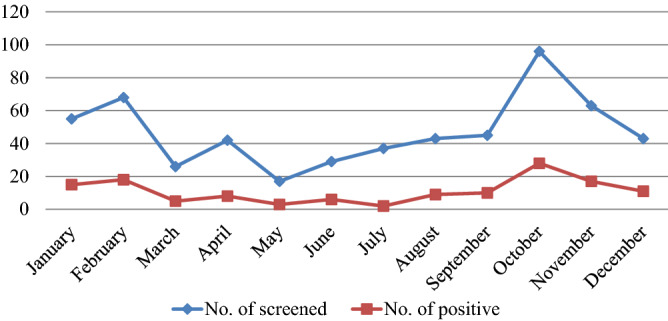
Visceral leishmaniasis patients’ distribution by months visiting at Tefera Hailu Memorial Hospital, Sekota, Northeast Ethiopia from January 2017 to December 2021.

Despite the rise and fall seen, VL cases were reported in almost every month and season of the year at Tefera Hailu Memorial Hospital. The highest number of confirmed cases was reported in October (28), followed by February (18). The distribution of the cases across the season showed that the maximum number of cases of VL (27.0% (55/204)) was registered in the autumn (September–November), followed by the winter (December-February) (26.5% (44/166)), while the least number of cases (18.8% (16/85)) were reported in the spring (March–May) (Fig. 3).

### Positivity rate of confirmed VL cases in relation to sex, age and residence

The majority of the 564 suspected VL patients were between the ages of 15 and 29, male, farmers, and rural residents. Likewise, the majority of VL confirmed participants were in the 15–29 year age group (37.1% (49/132))), males (78.0% (103/132)), farmers (47.7% (63/132)), and were rural residents (89.4% (118/132)) (Table 1).

**Table 1:** Socio-demographic characteristics of the study participants in relation to VL at Tefera Hailu Memorial Hospital, Sekota, NortheastEthiopia from January 2017 to December 2021.

Variables	No. of screened Number (%)	VL positive Number (%)	VL negative Number (%)	X2	P value
Age					
< 5	47 (8.3)	14 (29.8)	33 (70.2)		
5–14	87 (15.4)	23 (26.4)	64 (73.6)		
15–29	219 (38.8)	49 (22.4)	170 (77.6)	3.214	0.523
30–44	141 (25.0)	34 (24.1)	107 (75.9)		
≥ 45	70 (12.4)	12 (17.1)	58 (82.9)		
Sex					
Male	402 (71.3)	103 (25.6)	299(74.4)	13.839	0.031
Female	162 (28.7)	29 (17.9)	133(82.1)		
Occupation					
Pre school	72 (12.8)	16 (22.2)	56 (77.8)		
Student	102(18.1)	24 (23.5)	78 (76.5)		
Farmer	254(45.0)	63 (24.8)	191(75.2)	2.312	0.783
Housewife	59(10.5)	8 (13.6)	51 (86.4)		
Daily labourer	77(13.7)	21 (27.3)	56 (72.7)		
Residence					
Rural	421 (74.6)	118 (28.0)	303 (72.0)	19.807	< 0.001
Urban	143 (25.4)	14(9.8)	129 (90.2)		

**Figure 4:**
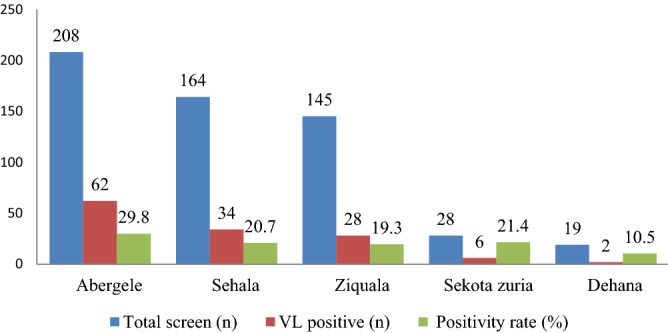
The geographical distribution of visceral leishmaniasis in the districts of Waghemra zone at Tefera Hailu Memorial Hospital, Sekota, Northeast Ethiopia from January 2017 to December 2021.

Among a total of eight districts in Waghemira Zone, cases were reported from five districts. Abergele district had the highest number of VL cases (47.0%) out of a total of 132, followed by Sehala (24.8%) and Ziquala (21.2%). However, the lowest number of VL cases were observed in Dehana district (1.5% (2/132)) (Fig. 4).

### Clinical profile abnormalities among visceral leishmaniasis patients

In this study, the common clinical signs and symptoms of VL patients were fever (96.2% (127/132)), splenomegaly (94.7% (125/132)), general weakness (80.3% (106/132)), skin mucosal pallor (77.3% (102/132)), weight loss (77.3% (102/132)), hepatomegaly (39.4% (52/132)), ascites (31.8% (42/132)) and jaundice (21.2% (28/132)) (Table 2).

**Table 2:** The frequency of clinical features of VL patients at Tefera Hailu Memorial Hospital, Sekota, Northeast Ethiopia from January 2017 to December 2021.

	Number (n)	Percentage (%)
Clinical symptoms		
Fever	127	96.2
Weight loss	102	77.3
Jaundice	28	21.2
Vomiting/diarrhea	21	15.9
Bleeding episodes	18	13.6
Abdominal pain	26	19.7
General weakness	106	80.3
Clinical signs		
Splenomegaly	125	94.7
Hepatomegaly	52	39.4
Skin mucosal pallor	102	77.3
Ascites	42	31.8
Palpable lymphadenopathy	8	6.1

**Figure 5:**
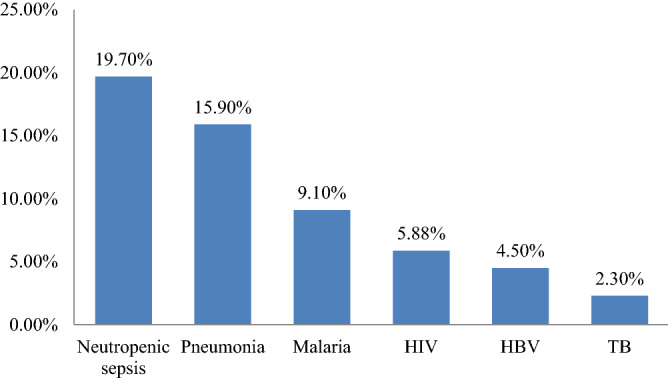
The rate of coinfection in VL patients attending at Tefera Hailu Memorial Hospital, Sekota, Northeast Ethiopia from January 2017 to December 2021.

Of the total, 48 VL patients (36.4%) had documented coinfections. Neutropenic sepsis was found to be the most common coinfection (19.7%) followed by pneumonia (15.9%) and malaria (9.1%). Moreover, from a total of 132 VL patients, 51.5% (68) of them were tested for HIV. Of the HIV tested, 5.9% (4/64) of patients were found to be co-infected with HIV (Fig. 5).

### Hematological parameters alteration in patients with visceral leishmaniasis

Based on the analysis, the absolute mean number of RBCs, level of hemoglobin, percentage of hematocrit, RBC indices, and absolute platelet counts were significantly lower in VL patients. Similarly, the absolute count of WBC, neutrophil count, lymphocyte count, and mixed (eosinophil and monocyte) count were also significantly lower in VL patients. In general, the most common hematological abnormalities observed in this study were anemia (86.4%), thrombocytopenia (81.8%), leucopenia (78.8%), neutropenia (74.2%), and pancytopenia (71.2%) (Table 3). Of the total 114 (86.4%) anemic patients, 54 (47.4%), 48 (42.1%), and 12 (10.5%) developed severe, mild, and moderate anemia, respectively.

**Table 3:** Hematological profiles of VL patients at Tefera Hailu Memorial Hospital, Sekota, Northeast Ethiopia from January 2017 to December 2021.

Parameters	Result	Normal	Low
	Mean ± Sd	Number (%)	Number (%)
Total WBCs (10^3^/μl)	2.42 ± 1.17	28 (21.2)	104 (78.8)
ANC (10^3^/μl)	1.25 ± 0.56	34 (25.8)	98 (74.2)
ALC (10^3^/μl)	0.85 ± 0.69	46 (34.8)	86 (65.2)
MXD (10^3^/μl)	0.2610 ± 0.24	44 (33.3)	88 (66.7)
RBCs (10^6^/μl)	3.03 ± 1.15	42 (31.8)	90 (68.2%)
Hgb (g/dl)	8.07 ± 3.10	18 (13.6)	114 (86.4)
HCT (%)	24.91 ± 8.50	25 (18.9)	107 (81.1)
MCV (fl)	76.8 ± 9.32	43 (32.6)	89 (67.4)
MCH (pg)	25.35 ± 4.35	76 (57.6)	56 (42.4)
MCHC (g/dl)	32.53 ± 2.60	122 (92.4)	10 (7.6)
RDW CV (%)	14.8 ± 3.2	84 (63.6)	Abnormal 48(36.4)
PLT (10^3^/μl)	93.46 ± 38.21	24 (18.2)	108 (81.8)
Pancytopenia		38 (28.8)	94 (71.2)

## Discussion

Visceral leishmaniasis is a major public health problem with high morbidity and mortality, especially in developing countries including Ethiopia^20^. In this study, 564 suspected VL patient records were reviewed at Tefera Hailu Memorial Hospital's leishmaniasis diagnostic and treatment center. The overall positivity rate for VL in this study was 23.4%. This indicates the endemicity and burden of VL in the Waghemra Zone. The finding was consistent with other similar retrospective studies conducted in Metema (22.6%)^21^ and West Armachiho district (21%)^22^, Northwest Ethiopia. However, it was lower than a study done in Addis Zemen, Ethiopia (39.1%)^23^. The observed variation might be due to differences in climate, altitude variation, study period, and community awareness of VL transmission and control.

The findings of this study revealed that the positivity rate of confirmed VL cases was showing a declining trend from 2017/18 (25.8%) to 2020/21 (20.9%). This declining trend of VL cases might be related to the proper implementation of VL prevention and control strategies like raising community awareness, increasing test availability, and making ITNs more accessible to the community^19^.

Regardless of the development of guidelines, Ethiopia currently lacks an effective leishmaniasis vector management program. Information regarding leishmaniasis is inadequate in developing countries, and surveillance systems for leishmaniasis are poorly established. Therefore, data collection and analysis for monitoring and evaluation of the program are crucial aspects^19^. Moreover, the distribution of insecticide-treated nets (ITNs) and insecticide spraying for malaria control may have some impact on Phlebotomine in lowland localities where VL is also endemic^24^.

The burdens of VL are largely determined by environmental, climatic, and seasonal variables^25^. In the study area, seasonal fluctuations in the VL positivity rate have been found. Autumn (September–December) has the highest number of VL cases, followed by summer (June–August). This was not in agreement with research conducted in Bahir Dar, Ethiopia^26^, which found that the highest positivity was reported in the winter (January–March), followed by the autumn. Seasonal fluctuations in rainfall and temperature affect the availability of sand fly vector breeding habitats, the length of larval development, and the rate of multiplication of Leishmania parasites inside the vector^27^. The transmission coincides with the major harvesting and planting seasons in the study area and in Ethiopian rural areas in general. This might negatively affect the economic development of the VL-endemic areas of the country^28,29^.

In terms of VL positivity rate by age group, the group 15–29 years old had the highest number of cases. This may be due to the fact that people in this age group are active and productive forces, as they are involved in agricultural and other activities that require them to travel to exposed places and spend the night outside to keep their products. This was consistent with a study conducted in Metema and West Armachiho, Northwest Ethiopia^21,22^. This study also discovered that males (78.0%) had a higher VL positivity rate than females (22.0%). This finding was in agreement with other studies conducted in various areas of Ethiopia^21,26,30^, Sudan^31^, and Kenya^32^. Males are expected to have a higher frequency of VL because they are more prone to engage in outdoor activities that expose them to sand fly bites^23,33^. Moreover, regarding their occupation, the majority (63, 47.7%) of the VL-confirmed patients were farmers, and 118 (89.4%) were from rural areas. This could be linked to sandfly breeding sites, and it's thought that the breeding sites are more concentrated in rural areas than in urban areas^34^.

Visceral leishmaniasis is a huge burden on healthcare facilities throughout the world, including Ethiopia. The disease is spreading, and new VL foci are now being reported in different regions of Ethiopia^29^. Because VL infection differs by geographical context and population, the control strategy must be according to the local epidemiology of each place^35^. The majority of VL cases found in this study were from Waghemra zone's poorest areas or districts, such as Abergele, Sehala, and Ziquala. A similar finding was reported in Nepal^36^. The association between poverty and hotspots reveals that VL is a disease that affects "the poorest of the poor," suggesting a potential role for waning immunity as an underlying driver of hotspots^37^.

On the other hand, the detection of VL cases in the highland districts of Waghemra Zone like Sekota Zuria, and Dehana might be due to the movement of people to the endemic areas. Similar findings were reported in a study done in Metema, and Bahirdar, Ethiopia^21,26^. Those seasonal migrants travel to the endemic areas for agricultural work, particularly harvesting during the summer, and manifest during the winter after the expected incubation period. The disease is spreading, and new endemic foci are now being reported in different countries, including the study area^22^. This study on VL risk area mapping could be valuable in identifying priority sites for establishing VL diagnosis and treatment services, as well as applying other prevention and control measures in the afflicted areas in the future. As a result, the national leishmaniasis control program should improve high-risk populations' access to VL prevention and prioritize high-risk areas based on stratification.

The major clinical symptom and sign presentations of VL patients were fever (96.2%), splenomegaly (94.7%), and general weakness (80.3%). Similar findings were observed in the study done in Bahir Dar^26^, Western Tigrai^38^, Ethiopia, Kenya^32^, and Northern Pakistan^39^. The development of fever and splenomegaly in leishmaniasis is possibly due to the release of many proinflammatory cytokines and the sequestration of red blood cells in the spleen^40^. Assessing the clinical presentations and laboratory profiles of visceral leishmaniasis is important for early diagnosis and the timely initiation of management.

A total of 48 (36.4%) VL patients had documented co-infections. Neutropenic sepsis and pneumonia were found to be the major co-infections, with a prevalence of 19.7% and 15.9%, respectively. This is due to the fact that VL causes pancytopenia and malnutrition; they are predisposed to different infections and bacterial sepsis. Sepsis is one of the predictors of death of patients with VL. Similar findings were reported in Bahir Dar, Ethiopia^26^, and Greece^41^. In this study also discovered that 9.1% of VL patients were also infected with malaria. This result was in line with the previous findings reported in Metema, Northwest Ethiopia^42^. Malaria and VL have regional overlap across East Africa, including Ethiopia, and their distribution is heavily impacted by environmental and behavioral variables, as well as the spread of biological insect vectors^43^.

From a total of 132 VL patients, 51.5% (68) of them were tested for HIV. Of the HIV tested, 5.9% (4/64) of patients were found to be co-infected with HIV. The HIV co-infection rate is lower than a study done in Bahir Dar, Ethiopia, which revealed 9.2% of HIV positives^26^ and 38% co-infection in the study from Northwest Ethiopia^44^. The low co-infection rate of HIV in this study may be due to the decreased incidence rate of HIV infection in the country. In addition, the test result was not documented in 64 patients, which might underestimate the co-infection rates. In this study, HIV testing among VL patients was so low (51.5%) compared to the national leishmaniasis guidelines recommendation that all VL patients should be screened for HIV and vice versa in endemic foci for VL^19^. This low level of HIV testing could be attributed to a shortage of HIV kits as well as a lack of awareness among healthcare providers.

Anemia (86.4%), thrombocytopenia (81.8%), leukopenia (788.8%), neutropenia (74.2%), and pancytopenia (71.2%) were the most common hematological abnormalities in this study. Similar findings were observed in various studies such as Gondar, Ethiopia^45^, Bahir Dar, Ethiopia^26^, Sudan^46^, Nepal^36^, Yemen^14^, and Iran^47^. The overall prevalence of anemia was 86.4%, which was in line with studies reported from Gondar, Ethiopia (85.5%)^48^ and Iran (83.8%)^47^. In contrast, this result was lower than those reported in studies from Gondar, Northwest Ethiopia (94.4% to 97.4%)^49^ and Kumaon region, India (93% to 100%)^50^, and Nepal (90%)^36^. Red blood cell (RBC) sequestration and destruction in an enlarged spleen, immunological processes, changes in RBC membrane permeability, and plasma volume increase could all contribute to anemia in these VL patients^51,52^. Regarding the severity of anemia, of the total 114 (86.4%) anemic patients, 54 (47.4%), 48 (42.1%), and 12 (10.5%) developed severe, mild, and moderate anemia, respectively. This finding was comparable with that reported from a study done in Western Tigrai, Ethiopia^38^.

The prevalence of thrombocytopenia in this study was 81.8%, which was consistent with studies done in North India (85%)^50^ and South India (83.3%)^53^. In contrast, it was lower than a study done in Gondar, Ethiopia (90.1%)^45^, Bahir Dar, Ethiopia (90.2%)^26^, and Iran (91.2%)^54^. However, the prevalence was higher than in Gondar, Ethiopia (75.8%)^48^ and Nepal (72.5%)^36^. Splenic sequestration and immune-mediated processes are regarded as the main causes of thrombocytopenia in VL patients^52^. The other hematological abnormality reported in this study was leucopenia, which was 78.8% prevalent. It was lower than a study done in Gondar, Ethiopia (95.4%)^45^, India (83.3%)^55^ and Yemen (87%)^14^. However, it was higher than studies done in Nepal (67.5%)^36^ and Iran (67.6%)^47^.

Neutropenia was one of the most common white blood cell abnormalities seen in 74.2% of VL patients in this study, which was similar to a study done in Gondar, Northwest Ethiopia (82.5%)^48^. The parasite might damage premature white blood cells, particularly neutrophils, resulting in a high prevalence of neutropenia^52^. Generally, pancytopenia was seen in 71.2% of VL patients. Similar findings were reported in Bahir Dar, Ethiopia (79.4%)^24^, and Yemen (72%)^14^. However, this finding was higher than a study done in Nepal (25%)^36^. Prior to the presentation, pancytopenia could be induced by long-term symptoms and splenomegaly, resulting in increased peripheral blood cell death^51^.

## Conclusion

In this study, the overall positivity rate of VL cases was 23.4% (132/564) and indicated a fluctuating yet decreasing trend in the recent year. This figure showed that VL is a substantial public health problem in the study area, affecting the productive segments of the population and occurring during key harvesting seasons, causing a detrimental impact on the local community. The highest numbers of VL cases were recorded in Abergele, Sehala, and Ziquala districts. As a result, the national leishmaniasis control program should improve high-risk populations' access to VL prevention and prioritize high-risk areas based on stratification. The majority of VL patients in this study had a fever, splenomegaly, general weakness, anemia, thrombocytopenia, and leucopenia. These clinical and hematological abnormalities are utilized to provide information to clinicians, which may help improve the management of VL patients by allowing for early diagnosis and prevention of clinical and hematological problems. Therefore, an elimination program that aims to combine case-finding, treatment, and vector control should be implemented in the study area. Moreover, a cohort study is needed to determine the risk factors for clinical and hematological abnormalities in VL patients.

## Data Availability

All data generated or analyzed during this study are included in this published article.
